# The Anti-Inflammatory, Anti-Apoptotic, and Antioxidant Effects of a Pomegranate-Peel Extract against Acrylamide-Induced Hepatotoxicity in Rats

**DOI:** 10.3390/life12020224

**Published:** 2022-01-31

**Authors:** Samy Sayed, Saqer S. Alotaibi, Ahmed M. El-Shehawi, Mohamed M. Hassan, Mustafa Shukry, Mohamed Alkafafy, Mohamed Mohamed Soliman

**Affiliations:** 1 Department of Science and Technology, University College-Ranyah, Taif University, P.O. Box 11099, Taif 21944, Saudi Arabia; 2 Department of Biotechnology, College of Science, Taif University, P.O. Box 11099, Taif 21944, Saudi Arabia; saqer@tu.edu.sa (S.S.A.); a.elshehawi@tu.edu.sa (A.M.E.-S.); m.kafafy@tu.edu.sa (M.A.); 3 Department of Biology, College of Science, Taif University, P.O. Box 11099, Taif 21944, Saudi Arabia; m.khyate@tu.edu.sa; 4 Department of Physiology, Faculty of Veterinary Medicine, Kafrelsheikh University, Kafrelsheikh 33516, Egypt; mostafa.ataa@vet.kfs.edu.eg; 5 Clinical Laboratory Sciences Department, Turabah University College, Taif University, Taif 21995, Saudi Arabia

**Keywords:** a pomegranate-peel extract, acrylamide, hepatotoxicity, antioxidant, anti-apoptotic

## Abstract

The Acrylamide is a toxic compound generated under oxidative stress arising from intracellular ROS production and induced toxicity. It is frequently used in industry and generated through the heating of tobacco and foods high in carbohydrates. The exact mechanism of its toxicity is still unclear. In this study, an extract of the peels of pomegranate (*Punica granatum* L.), a nutritious and visually appealing fruit with a diverse bioactive profile, was examined for its potential anti-apoptotic, antioxidant, and anti-inflammatory effects. A total of 40 adult male Wistar rats were allocated into four groups of 10 rats each: Group 1 was a negative-control group (CNT) and received normal saline; Group 2 was a positive-control acrylamide group and received acrylamide orally at a dose of 20 mg/kg/bw; in Group 3, the rats were supplemented with pomegranate-peel extract (P.P; 150 mg/kg/bw) orally on a daily basis for 3 weeks, administered simultaneously with the acrylamide treatment described for Group 2; Group 4 was a protective group, and the animals received the pomegranate-peel extract and acrylamide as stated for Groups 2 and 3, with the pomegranate-peel extract (P.P. extract) administered 1 week earlier than the acrylamide. The results indicate that acrylamide exposure increased the serum levels of AST, ALT, creatinine, interleukin-1 beta, and interleukin-6 in an extraordinary manner. In addition, it increased the lipid peroxidation marker malondialdehyde (MDA) and simultaneously weakened antioxidant biomarker activities (SOD, GSH, and catalase) and reduced the levels of interleukin-10. The pomegranate-peel extract was shown to reduce the inflammatory blood markers of interleukin-1 beta and IL-6. Glutathione peroxidase, superoxide dismutase, catalase, and interleukin-10 were all significantly elevated in comparison to the acrylamide-treatment group as a result of the significant reduction in MDA levels induced by the P.P extract. In addition, the pomegranate-peel extract normalized the cyclooxygenase-2 (COX2), transforming growth factor-beta 1 (TGF-β1), and caspase-3 levels, with a significant upregulation of the mRNA expression of heme oxygenase-1 (HO-1), nuclear factor erythroid 2 (Nrf2), and Bcl-2. Therefore, these data reveal that pomegranate peel has anti-inflammatory, antiapoptotic, free-radical-scavenging, and powerful antioxidant activity that protects against acrylamide toxicity.

## 1. Introduction

Acrylamide is a transparent, colourless, odourless, crystallized monomer [1]. Glucose and asparagine react to form this product [2]. Acrylamide is abundant in tobacco smoke and also contained in bread, crisps/potato chips, French fries, and coffee. Its polymer, polyacrylamide, is utilized in industries such as textiles, wastewater treatment, paper mining, and cosmetics [3].

Acrylamide can be absorbed through the digestive system, the respiratory system, or the skin [4]. Due to its low molecular weight and high water absorption, organic barriers such as the blood–placenta and blood–milk barriers permit the passage of acrylamide [5]. Nucleic acids, vinyl groups, and NH2 and SH moieties in proteins work together to allow acrylamide to cross biological membranes [6], resulting in neurotoxicity, genotoxicity, and malignant growth in mammals [1].

Oxidative stress is the cause of acrylamide’s harmfulness. The metabolization of acrylamide by glutathione or cytochrome P450 to glicidiamine is a greater danger than acrylamide itself [7]. The formation of glicidiamine is prevented by exogenous antioxidants, which guard against oxidative damage [8]. High doses of acrylamide change the oxidative status and enzyme activity, dramatically increasing oxidative stress [9].

One of the top seven fruits for human health is the pomegranate (*Punica granatum* L.) [10]. Thyroid disorders, asthma, and diarrhoea are just some of the health conditions that can be treated or prevented by consuming pomegranates [11]. It is well-known that pomegranate fruit contains ellagic acid and other hydrolysable tannins. The juice contains anthocyanins, mainly cyanidin and delphinidin glucosides, but these pigments can also be found in the arils and peel at different levels [12]. Simple sugars (30–35%), phenolic compounds (10–20%), and polysaccharides make up the majority of the dry peel, whereas the fresh peel has a high water content of around 70–75% [13]. Pomegranate-peel extract shows a high antioxidant activity in haemodialysis patients and it reduces the toxicity of acetaminophen in the liver [14,15].

Pomegranate juice has a wide range of health benefits, including antioxidant activity [16,17,18,19,20,21,22,23,24,25,26,27], antimutagenic activity [19], antiviral activity [28], anti-obesity [29,30], anti-cryptosporidial [31], as well as the treatment of neurodegenerative illnesses [32]. Moreover, pomegranate peel showed antioxidant activity [20,24,33,34] and antimicrobial activity [35]. Furthermore, Pomegranate seed extract was found to have antioxidant properties in several studies. [20,24,36,37].

Taif is a high-altitude location with elevations ranging from 1200 to 2300 m above sea level, making it ideal for a diverse range of indigenous plant species [38]. Grape and pomegranate (*Punica granatum Lythraceae*) are two of the most important natural fruit trees in Taif. Taify pomegranate is the most widely grown pomegranate type in Saudi Arabia. It has a significant cultural, nutritional, and commercial importance [29]. As a result of Taif’s altitude elevation (natural home for Taify pomegranate), pomegranate could supply a unique source of antioxidants [29,39].

Given this background, we performed an experiment to investigate the hepatotoxicity of acrylamide, and to assess the protective effect of pomegranate extract and its intracellular pathway against the toxicity of acrylamide.

## 2. Materials and Methods

### 2.1. Animals and Experiments

Our experiment involved 40 male Wistar rats which were 10 weeks old. The rats had free access to food and water and were housed at room temperature. Over the course of seven days, the rats were handled manually in the Turabah University labs to adapt them to the experimental conditions. Four groups were established, with 10 rats per group. Group 1 was a negative-control group (CNT), which received normal saline; Group 2 was a positive-control acrylamide group, which was orally administered acrylamide at a dose of 20 mg/kg/bw [40,41] for 2 weeks. This is considered to be the optimal dose that induces total toxicity for the liver [42]. In group 3 (P.P.), the rats were orally administered pomegranate-peel extract (150 mg/kg/bw) daily for 3 weeks [43]; Group 4 was a protective group that received the pomegranate-peel extract (P.P.) and acrylamide as stated for groups 2 and 3, but the pomegranate-peel extract was given 1 week before the beginning of the acrylamide treatment.

On Day 21, all of the rats were anesthetized using isoflurane and decapitated. Blood samples and liver tissues were harvested under aseptic conditions. The serum was extracted, used for chemical analysis, and stored at −20 °C. Hepatic tissue samples for RNA analysis and real-time PCR were preserved in QIAzol reagent. Bouin solution was used to preserve liver samples for histology and immunohistochemistry.

### 2.2. Plant Samples and Extraction

In October 2020, fresh pomegranate fruits were collected from a farm in the Al-Hada region of Saudi Arabia’s Taif region. The peels were air-dried before being ground into a fine powder. The fine powder was extracted for 3 days at room temperature with 1000 mL of absolute ethanol. The extract was centrifuged at 7000 rpm for 15 min and filtered 3 times with Whatman filter paper No.1 to obtain a pure supernatant. The supernatant was evaporated at 30 °C using a Buchner funnel in a rotary vacuum evaporator. The pellet was then dissolved in a 1% aqueous solution of dimethyl sulfoxide (DMSO). The extract was kept at 4 °C until the HPLC analysis and experiments were used.

### 2.3. HPLC Analysis for Phenol and Flavonoid Compounds

**Figure 1:**
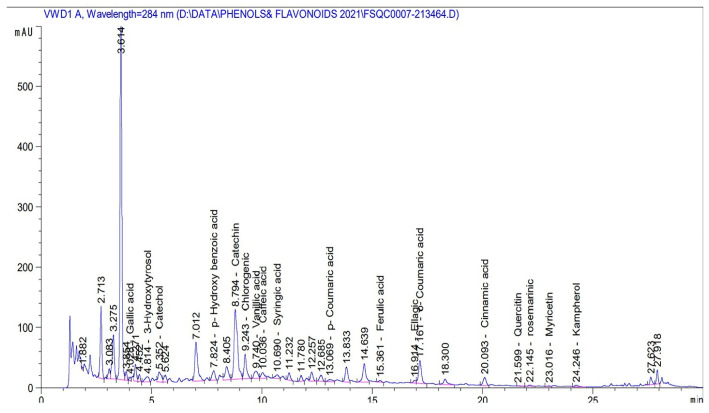
HPLC analysis for phenol and flavonoid compounds.

The extract was analysed, and phenol and flavonoid compounds were detected according to the protocol [44], where an Agilent 1260 Infinity HPLC (Agilent, USA) was utilized, equipped with a quaternary pump. A Kinetex^®^ 5 µm EVO C18 100 mm × 4.6 mm (Phenomenex, Santa Clara, CA, USA) column was used, operated at 30 °C. The separation was conducted using a ternary linear elution gradient with (A) HPLC-grade water and 0.2% H_3_PO_4_ (*v*/*v*), (B) methanol, and (C) acetonitrile. Then, 20 µL of the sample was injected, and a variable wavelength detector set at 284 nm was used to recognize phenols and flavonoids. HPLC is selected to convert each component’s area under the curve compared to the area under the standards curve to a concentration (mg/L). In the case of making diluted samples, the concentration is multiplied by the dilution factor. The work was conducted at Cairo University’s Faculty of Agriculture’s Food Safety and Quality Control Laboratory (Reference Number: FSQC0007-21). See Table 1 and Figure 1.

The concentration of phenols and flavonoids are expressed on mg/L. mAu is milli-absorbance unite.

### 2.4. Blood Parameter Measurement

The serum levels of ALT, AST, and urea were measured using a colorimetric spectrophotometer as described in the instruction manual. The procedures described in [45] were used to assess malondialdehyde (MDA). The levels of superoxide dismutase (SOD), reduced glutathione (GSH), and catalase, as well as total proteins, were measured using previously established methods [46,47]. A spectrophotometer set at 412 nm was used to determine GSH following the Tietze method [48].

### 2.5. Measurement of Inflammatory and Anti-Inflammatory Cytokines

The ELISA kits for rat IL-1β and IL-6 (ab100768 and ab234570, respectively) were used with an ELISA spectrophotometer as described in the instruction manual for each kit. IL-10 was measured using a commercial kit available from Abcam co. USA (Rat IL-10 ELISA Kit (ab100765)). The levels were calculated based on data obtained from the ELISA reader according to the instructions provided for each parameter.

### 2.6. Quantification of Genes by Real-Time PCR (qRT-PCR)

Total RNA was extracted from the liver samples and quantified using a spectrophotometer at 260 nm according to the manufacturer’s instructions (BIORAD, Hercules, CA, USA). To make cDNA, two grams of isolated RNA was used (MyTaq Red Mix, Memphis, TN, USA). To quantify the expression of different hepatic genes, synthesised cDNA was amplified with SYBR Green master mix (Thermo Scientific, Waltham, MA, USA). Table 2 lists the quantification primers used in this study. The CFX96 Touch™ Real-Time PCR system (Bio-Rad Co., Hercules, CA, USA) was used, and the data were analysed using the 2−ΔΔCt method. Comparative cycle threshold (CT) values standardised to beta-actin were used to measure the changes in gene intensity and expression.

### 2.7. Histopathological Examination

Hepatic tissue samples were fixed in buffered formalin with a neutral pH (10%). Liver samples were processed and stained with haematoxylin and eosin [49].

### 2.8. Immunohistochemical Study

The immunohistochemical study was conducted following the method of [50]. Tissue sections were cleaned of wax and rinsed in 0.05 M citrate buffer, pH 6.8. Non-specific binding was prevented by using 0.3% H2O2 and a protein blocker on the sections. The sections were incubated with a rabbit monoclonal primary antibody (anti-bcl2, Abcam, Cat# ab182858, dilution 1:500). After being washed in phosphate-buffered saline, the sections were incubated with a goat anti-rabbit secondary antibody ((EnVision System Horseradish Peroxidase Labelled Polymer; 3 Fountain Drive Inchinnan Business Park Paisley PA4 9RF, UK) at room temperature for 30 min. A DAB kit was used to visualise the slides, and Mayer’s haematoxylin was used to counterstain them. The Bcl2 immunolabelling indices are expressed as percentages of positive expression in 1000 cells per 8 high-power fields (HPFs).

### 2.9. Statistical Analysis

Data are represented as the means ± SEs. The results were evaluated using one-way analysis of variance (ANOVA). For the comparison of significance between the groups, Duncan’s test was utilized as a post hoc test, and *p*-values less than 0.05 were considered to indicate statistical significance.

## 3. Results

### 3.1. Impacts of Pomegranate-Peel Extract on Acrylamide-Induced Liver Dysfunction in Rats

Our results indicated that there were noteworthy increases (*p* < 0.05) in urea levels, accompanied by decreased total protein levels, in acrylamide-treated rats in relation to the other treatment groups. These effects were overcome in the rats that were simultaneously treated with pomegranate-peel extract (Group 3). In addition, Table 3 indicates that there were substantial increases (*p* < 0.05) in the AST and ALT levels in acrylamide-treated rats in relation to the other treatment groups. The rats co-treated with P.P. extract and acrylamide showed a significant normalization of both AST and ALT levels.

### 3.2. Impacts of Pomegranate-Peel Extract on Acrylamide-Induced Alterations of Serum MDA, Catalase, GSH and SOD Levels

Table 4 shows that there were significant decreases in the catalase, SOD, and GSH levels, with significant (*p* < 0.05) increases in the MDA level in acrylamide-treated rats in relation to the other treatment groups. The rats co-treated with P.P. extract and acrylamide showed a significant normalization of catalase, SOD, and GSH levels, with significant decreases in the MDA levels in relation to the acrylamide-treated rats.

### 3.3. Impacts of Pomegranate-Peel Extract on Acrylamide-Induced Alterations in Cytokine Levels

Table 5 shows that there was a substantial increase (*p* < 0.05) in the levels of IL-1β and IL-6 in acrylamide-treated rats in comparison to the other treatment groups. IL-1β and IL-6 levels were significantly reduced in the rats co-treated with P.P. extract and acrylamide in comparison to the acrylamide-treated rats. Our data concerning IL-10 reveal that there was a significant decrease in IL-10 levels in the acrylamide-treated rats, which showed a significant enhancement in the rats co-treated with P.P. extract and acrylamide.

### 3.4. Impacts of Pomegranate-Peel Extract on the Expression of Liver Genes in Acrylamide-Induced Liver Dysfunction in Rats

**Figure 2:**
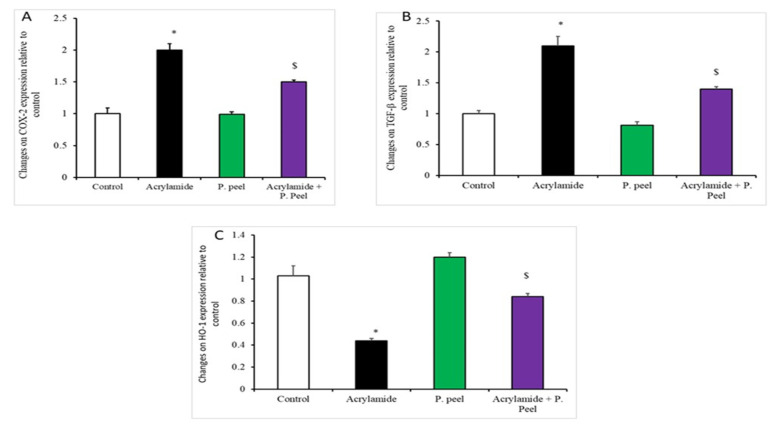
Impact of pomegranate-peel extract on the expression of liver genes in acrylamide-induced liver dysfunction in rats. A. COX2; B. TGF-β; C. HO-1. Values are means ± SEMs for 10 different rats per experiment. Values are statistically significant at * *p* < 0.05 vs. control and P.P. extract groups and $ *p* < 0.05 vs. the acrylamide group. P.P., pomegranate peel *n* = 10.

**Figure 3:**
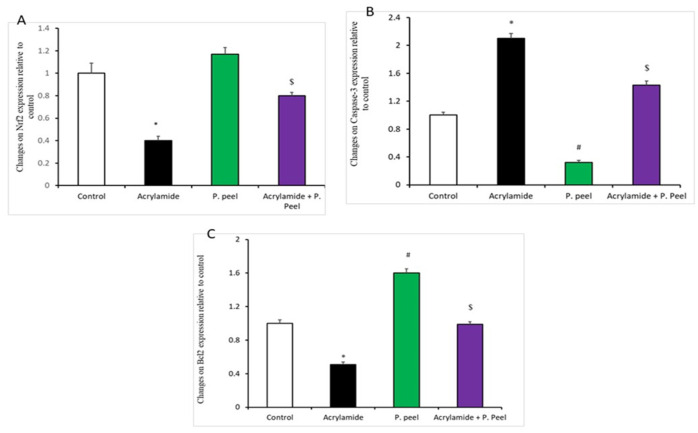
Impact of pomegranate-peel extract on the expression of liver genes in acrylamide-induced liver dysfunction in rats: A. Nrf2. B. Caspase-3. C. BCL-2. Values are means ± SEMs for 10 different rats per group. Values are statistically significant at * *p* < 0.05 vs. control and P.P. extract groups and $ *p* < 0.05 vs. the acrylamide group., # *p* < 0.05 vs. all other three tested groups. P.P., pomegranate peel *n* = 10.

Figure 2 and Figure 3 indicate that there was a significant upregulation of the mRNA expression of COX2, TGF-B1, and caspase-3 in the acrylamide-treated rats in relation to the other treatment groups; such expression was significantly decreased in the rats co-treated with P.P. extract and acrylamide in comparison to the acrylamide-treated rats. In addition, there was a noteworthy downregulation of the mRNA expression of HO-1, Nrf2, and Bcl2 in the acrylamide-treated rats in relation to the other treatment groups. Rats co-treated with P.P. extract and acrylamide showed a significant upregulation of the mRNA expression of HO-1, Nrf2, and Bcl2 in comparison to the acrylamide-treated rats.

### 3.5. Impacts of Pomegranate-Peel Extract on the Liver Architecture (Immunohistochemical and Histopathological Study)

**Figure 4:**
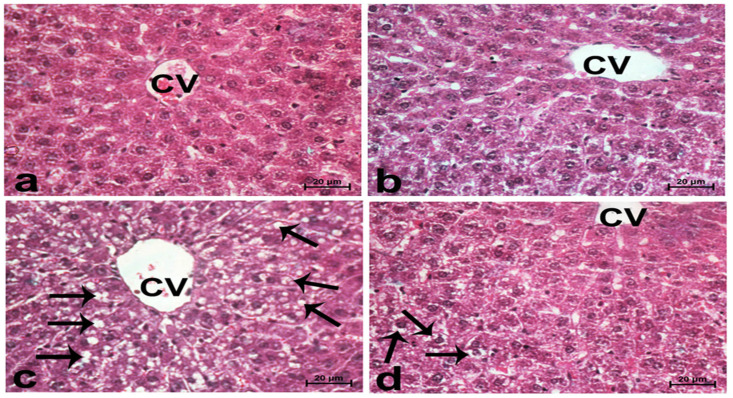
Photomicrographs of rat liver sections stained with haematoxylin and eosin in the control group (**a**), the P.P. extract-treated group (**b**), the acrylamide-treated group (**c**), and the group co-treated with acrylamide and pomegranate-peel extract (**d**). Sections from normal rats (**a**,**b**) displayed normal features of hepatic parenchyma organized in hepatic cords radiating from the central veins (CVs) at the centre of the hepatic lobule towards the lobular periphery. Sections from acrylamide-treated rats (**c**) showed degenerative changes as indicated from vacuolar hepatocytes (arrows). Rats co-treated with acrylamide and pomegranate juice (**d**) had reduced degenerative changes. Scale bar = 20 µm (original magnification = 400×).

**Figure 5:**
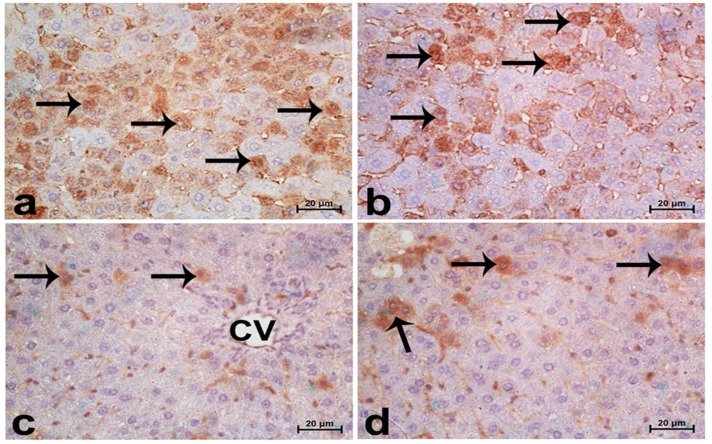
Photomicrographs of rat hepatic sections immunostained with Bcl2 antibody: control group (**a**), P.P. extract-treated group (**b**), acrylamide-treated group (**c**), and the group co-treated with acrylamide and pomegranate-peel extract (**d**). The intensity of immunostaining was remarkable in control (**a**) and pomegranate-peel extract (**b**) groups, as indicated from the strongly positive hepatocytes (arrows); it was reduced in the acrylamide-treated group (**c**) and was restored to moderate levels in co-treated rats (**d**). The reactive liver cells (arrows) were usually next to central veins (CVs). Reactive hepatocytes are marked with arrows. Scale bar = 20 µm (original magnification = 400×).

Sections from normal rats displayed normal features of hepatic parenchyma organized in hepatic cords radiating from the central veins. Sections from acrylamide-treated rats (Figure 4c) showed degenerative changes, as indicated from the vacuolar hepatocytes. Rats co-treated with acrylamide and pomegranate peel extract (Figure 4d) displayed lesser degenerative changes. Regarding the photomicrographs of rat hepatic sections immunostained with the Bcl2 antibody (Figure 5), the intensity of immunostaining was remarkable in the control and pomegranate peel extract-treated groups and reduced in the acrylamide group (Figure 5c), while it restored to moderate levels in the co-treated rats (Figure 5d).

## 4. Discussion

Reactive oxygen species (ROS) are a by-product of the natural metabolism of oxygen in mammalian bodies, and are neutralized by the antioxidant system [51]. Increased ROS production due to exposure to toxic chemicals and xenobiotics leads to an imbalance between the production of ROS and their removal by antioxidant systems [52]. The increased ROS attack cell membranes and damage biomolecules such as proteins and DNA. Another factor contributing to the development of diseases such as asthma, atherosclerosis, cancer, and diabetes is a weakening of the body’s natural antioxidant defences [53]. The toxicity of acrylamides is mainly dependent on ROS production [54].

In both healthy and diseased states, the liver is responsible for the organized metabolism of a variety of nutrients, including carbohydrates and toxins [55]. The purpose of the present study was to determine the protective effect of pomegranate-peel extract against acrylamide-induced hepatic damage. In our study, the acrylamide-exposed group showed a significant elevation in liver enzyme activities, indicating hepatic injury. Our data corroborated those of a preceding study, which found that acrylamide significantly increased AST and ALT levels [56]. Liver cell membranes may be damaged by glycidamide when it reacts with the functional groups of membrane proteins, resulting in specific lesions that affect permeability [57]. Many tissues contain AST and ALT. ALT is a more specific marker of liver damage and a more specific enzyme in the liver parenchyma. ALT is a biomarker for membrane damage, and AST is a biomarker for mitochondrial damage. ALT and AST are released into the bloodstream by damaged liver cells during hepatotoxicity [58]. Our results are in agreement with those of Gedik et al. [59], who reported that the hepatic toxicity induced by acrylamides resulted in marked increases in liver enzymes due to liver damage.

As confirmed by the reduced histopathological damage, the pomegranate-peel extract meaningfully restored the liver markers. This is consistent with the results in [60], where the authors revealed that oxidative-stress-induced liver injury could be reduced by pomegranate’s antioxidant properties. In addition, they confirmed that the acrylamide toxicity was found to have significantly decreased on the basis of the determined protein levels as well as elevations in serum urea levels. According to previous studies, acute acrylamide administration causes renal dysfunction [61]. This effect was restored by the pomegranate treatment. Pomegranate phenolic compounds have been found to possess antioxidant and free-radical-scavenging properties [62] and the kidney weight/body weight ratio significantly increased [63].

Acrylamides have been found to disrupt the oxidant/antioxidant balance, leading to an increase in ROS and, ultimately, oxidative injury [64]. In the endogenous antioxidant stress defence system, enzymes significantly contribute to the fight against the potentially harmful effects of hydrogen peroxide and lipid peroxidation. In line with the research findings of Zhao et al. [65], a reduction in antioxidant activities was found in the rats exposed to acrylamides in this study. After acrylamide treatment, the levels of the oxidative stress biomarker MDA significantly rose. Several other recent studies have found similar results [41], suggesting that acrylamides are detrimental because they increase lipid peroxidation and alter antioxidant enzyme systems, having negative effects on tissues. In the present study, SOD, GSH-Px, and CAT activity was decreased, while MDA levels were observed to be substantially elevated, with acrylamide treatment. 

In our study, pomegranate peel extract administration, alone or in combination with acrylamides, significantly increased the levels of SOD, GSH, and CAT, and decreased the levels of MDA in the blood. These findings agree with those in [66,67], where the authors proved that pomegranate-peel extract reduced MDA levels and increased GSH-Px activities, supporting the protective impact of pomegranate-peel extract on liver function and oxidative markers. In addition, our findings are in line with [68], who found that pomegranate-peel-extract supplementation reduced liver oxidative injury and improved hepatic structure and function in rats with bile duct ligation. The antioxidant enzymes were meaningfully augmented in response to pomegranate-peel-extract treatment in carbon-tetrachloride-induced liver injury [69]. Treatment with pomegranate-peel extract resulted in an increase in GSH levels, which may help to protect tissue from oxidative damage. In the GSH redox cycle, the activities of GSH, glutathione reductase, and glutathione peroxidase control the spread of free-radical reactions that cause lipid peroxidation [70].

Pomegranate (*Punica granatum* L.) is high in natural antioxidants (anthocyanins, catechins, quercetin, gallotannins, ellagitannins, ellagic, ferulic, and gallic acid), which have promising antioxidant properties [71]. Ellagitannins (punicalagin and its derivatives) are the most abundant polyphenols in the peel, and they are responsible for the peel’s potent antioxidant properties.

Pomegranate’s more excellent antioxidant activity is due to the inclusion of several phenolic components (ellagic acid, punicalin, and punicalagin) instead of single pure polyphenols [72]. Pomegranate methanolic fraction (at 80 g/mL concentration) had a high level of ellagitannins and had the most increased total antioxidant activity (5067.7 mol ascorbic acid equivalents [AAE]/g) when tested using the phosphomolybdenum technique [73]. Due to its outstanding efficiency in scavenging hydroxyl and superoxide anion radicals, pomegranate was a good source of natural antioxidants [74].

In DPPH radical scavenging, superoxide radical scavenging, and lipid peroxidation inhibitory experiments, carpellary membrane extract from pomegranate fruit showed a vigorous antioxidant activity [75]. Ellagic acid and gallic acid are powerful free radical scavengers found in PoP that help restore hepatic enzyme activity (catalase, peroxidase, and superoxide dismutaseas) while also suppressing lipid peroxidation [76]. Due to its higher ability to heal intestinal damage and decrease the expression of intestinal pro-inflammatory factors, elagic acid from pomegranates produced more protective effects than punicalin and punicalagin [77]. Intestinal motility and fluid accumulation are inhibited by the tannins and flavonoids in aqueous Pomegranate, making it practical for gastrointestinal ailments [78]. Lee, et al. [79] the anti-inflammatory properties of four hydrolyzable tannins extracted from Pomegranate (punicalagin, punicalin, strictinin A, and granatin B) were studied, and it was discovered that granatin B was a potent anti-inflammatory molecule in Pomegranate. Punicalagin-rich pomegranates inhibited chronic intestinal inflammation in Caco-2 cells, an in vitro model of the human intestinal epithelium, by directly molecular trapping pro-inflammatory gene transcription and protein levels [80].

The cellular metabolism of cytokines relies on the liver’s ability to synthesize and eliminate them [81]. Chronic liver disease raises interleukin-1b, interleukin-6, TNF-a, and interferon-gamma [82]. Our data revealed that there were significant increases in IL-1β and IL-6 in the acrylamide-treated rats in comparison to the other treatment groups; this result is consistent with the results reported before [83], where the authors reported that acrylamide exposure significantly increased the serum levels of interleukin-1β, interleukin-6, and TNF-α. In addition, the authors in [84] found increased IL-1 and TNF-α levels, indicating that acrylamide exposure causes inflammation in brain tissues.

Several studies have shown that pro-inflammatory cytokines such as TNF-α, IFN-β, and IL-6 are increased by free-radical-mediated apoptotic mechanisms. It seems to be reasonable to hypothesise that antioxidants and free-radical scavengers could counteract this effect, which supports our findings indicating that co-treatment with pomegranate-peel extract and acrylamide overcame the effects of acrylamide concerning the release of cytokines. In addition, in a study by Toklu et al., treatment with pomegranate-peel extract reduced the levels of TNF-α and IL-1β in BDL rats, indicating that pomegranate peel reduces the oxidative liver damage caused by BDL via its antioxidant effects [68]. Additionally, the antioxidant properties of pomegranate juice have been found to play an important role in modulating inflammatory cell signalling in colon cancer cells, attributable to its polyphenolic phytochemicals [85]. Pomegranate fruit has also been found to inhibit mitogen-activated protein (MAP) kinase-induced IL-1β indicating that pomegranate juice may prevent inflammatory responses that cause oxidative damage [86].

Our data indicate that there was a significant decrease in the IL-10 levels in the acrylamide-treated rats, while significantly higher levels were observed in the rats co-treated with P.P. extract and acrylamide; these results agree with those of [87], where the authors found that the acrylamide group had a meaningfully lower level of the anti-inflammatory IL-10. In the same manner, in [88], pomegranate peel extract was found to increase IL-10 levels in the brain. These results are valuable, as one of the most important mediators of cerebral I/R recovery is the anti-inflammatory cytokine IL-10 [89]. IL-10 reduces tissue inflammation by inhibiting the release of many inflammatory cytokines and chemokines, or by blocking the activities of TNF-α [90].

The inflammatory process is modulated by NF-κB, resulting in increases in inflammatory cytokines such as IL-1β, IL-6, and COX2 [91]. Our results show that the mRNA expression of COX2, TGF-β1, and caspase-3 was pointedly upregulated in acrylamide-treated rats in comparison to the other groups, but this expression was significantly decreased in the rats co-treated with P.P. extract and acrylamide. These findings are supported by other studies [92,93] stating that acrylamides induce NF-κB and that neuroinflammation occurs due to the upregulation of the pro-inflammatory cytokine. In addition, when TGF-β1 is present, it is known to induce apoptosis in a wide range of cell types [94], and it is strongly increased in chronic diseases [95]. Regarding the role of other cytokines, Li et al. [96] reported that caspase-3 expression in the spinal cord and sciatic nerves of acrylamide-treated rats was significantly elevated; this is in agreement with our findings and supports our results concerning the downregulation of Bcl-2 mRNA expression in acrylamide-treated rats. Acrylamide treatment has been linked to a reduction in Bcl2 and an increase in caspase-3 in the rat brain and cerebellum [97]. The downregulation of Bcl2 expression and the subsequent increase in caspase-3 activity causes apoptosis. In addition, we observed a significant downregulation of the mRNA expression of HO-1, Nrf2, and Bcl2 in acrylamide-treated rats in relation to the other treatment groups, while rats co-treated with P.P. extract and acrylamide showed a significant upregulation of the expression of these mRNAs. These results are supported by those in [98], where the authors showed that sulforaphane co-treatment activated the Nrf2 signalling pathway, protecting against acrylamide-induced neurotoxicity. In addition, our obtained results revealed that the intensity of Bcl2 immunostaining was remarkable in the control and pomegranate peel extract-treated groups, strengthening our finding that Nuclear factor-erythroid factor 2 (Nrf2) is a key target for preventing acrylamide’s toxicity. The protective intracellular pathway of pomegranate-peel extract proceeded through the modulation of apoptosis and cytokine production, and through the Nrf2/HO-1 pathway, as confirmed in [99]. The authors reported that pomegranate-peel powder inhibited the expression of the TNF-α and COX2 genes in the gastric mucosa, supporting the anti-inflammatory effects of the pomegranate. In addition, the authors in [67] reported that pomegranate-peel extract reduced liver fibrosis in rats by suppressing the p38MAPK/Nrf2 pathway. Genes protecting against oxidative stress are activated when Nrf2 detects an increase in oxidative stress [100]. One study found that CCl_4_-induced liver fibrosis may be alleviated by actuating Nrf2, which is consistent with our findings [101]. Finally, our obtained results are supported by Wei et al. [67], who reported that these protective effects could be attributed to lower levels of TGF-β1 and MDA, and increased GSH-Px activity, Nrf2 and HO-1 expression, implying that antioxidative activity could be a possible mechanism of pomegranate’s protection against acrylamide toxicity.

## 5. Conclusions

This is the first study to describe, in detail, the hepatic toxicity of acrylamides in conjunction with a description of the possible ameliorative effects of pomegranate-peel extract. Our results indicate that pomegranate effectively reduced the toxicity of acrylamides in the treated rats. A possible mechanism for niaction may be related to the improvement of liver function; reduction of oxidative stress and inflammatory responses; and modulation of COX2, TGF-B1, and caspase-3, along with a significant upregulation of the expression of HO-1 in rats. Thus, our results suggest that pomegranate-peel-extract supplementation can reduce the toxicity of acrylamides.

## Figures and Tables

**Table 1:** Components of phenols and flavonoids in the pomegranate peel tested extracts.

S.N.	Compounds	Retention Time (min)	Amount	Area (mAU*s)
1	Gallic acid	4.028	0.96	28.84930
2	3-Hydroxytyrosol	4.814	36.34	102.20006
3	Catechol	5.352	18.12	164.68491
4	p- Hydroxy benzoic acid	7.824	15.68	148.26949
5	Catechin	8.794	21.04	1268.24951
6	Chlorogenic acid	9.243	11.65	317.87186
7	Vanillic acid	9.740	8.63	138.28169
8	Caffeic acid	10.036	2.04	101.89981
9	Syringic acid	10.690	2.03	69.65744
10	p- Coumaric acid	13.069	0.71	48.37119
11	Ferulic acid	15.361	0.68	25.69361
12	Ellagic acid	16.914	1.50	38.31996
13	o- Coumaric acid	17.161	4.95	335.75812
14	Cinnamic acid	20.093	1.89	126.55923
15	Quercitin	21.599	2.35	4.63419
16	Rosemarinic acid	22.145	11.31	19.64437
17	Myricetin	23.016	5.60	9.34549
18	Kampherol	24.246	7.99	30.73397
Total			153.51	

**Table 2:** Primer’s sequence used for quantitative real-time PCR in liver and kidney of rats.

Gene	Direction	Primer Sequence	Accession Number
TGF-β1	Sense	GGACTACTACGCCAAAGAAG	NM_021578.2
	Antisense	TCAAAAGACAGCCACTCAGG	
COX2	Sense	TGATCTACCCTCCCCACGTC	NM 017232
	Antisense	ACACACTCTGTTGTGCTCCC	
BAX	Sense	AGGACGCATCCACCAAGAAG	NM 017059
	Antisense	CAGTTGAAGTTGCCGTCTGC	
Nrf2	Sense	TTGTAGATGACCATGAGTCGC	NM_031789.2
	Antisense	TGTCCTGCTGTATGCTGCTT	
HO-1	Sense	GTAAATGCAGTGTTGGCCCC	NM_012580.2
	Antisense	ATGTGCCAGGCATCTCCTTC	
Bcl2	Sense	ACTCTTCAGGGATGGGGTGA	NM_016993
	Antisense	TGACATCTCCCTGTTGACGC	
β-actin	Sense	AGGAGTACGATGAGTCCGGC	NM 031144
	Antisense	CGCAGCTCAGTAACAGTCCG	

TGF-β1; Transforming growth factor beta 1., COX2; Cyclooxygenase, Bax, Bcl-2-associated X protein. Bcl-2, B-cell lymphoma 2. Nrf2. The nuclear factor erythroid 2-related factor 2 (Nrf2), HO-1; Heme oxygenase-1.

**Table 3:** Protective impacts of pomegranate-peel extract on acrylamide-induced liver dysfunction in rats.

	Control	Acrylamide	P.P.	P.P. + Acrylamide
Urea (mg/dL)	16.8 ± 1.1 ^d^	58.1 ± 1.9 ^a^	22.9 ± 2.9 ^c^	39.8 ± 2.2 ^b^
Total proteins (g/dL)	9.1 ± 0.4 ^a^	4.9 ± 0.1 ^c^	9.1 ± 0.5 ^a^	8.9 ± 0.3 ^b^
AST (U/L)	31.7 ± 2.6 ^c^	149.7 ± 10.8 ^a^	29.3 ± 1.58 ^c^	71.8 ± 1.6 ^b^
ALT(U/L)	32.1 ± 1.1 ^c^	135.1 ± 3.7 ^a^	30.7 ± 1.1 ^c^	52.1 ± 2.1 ^b^

Means within the same row carrying different superscript letters a, b, c, d are significantly different at *p* < 0.05.

**Table 4:** Protective effects of pomegranate-peel extract against acrylamide-induced alterations in serum MDA, catalase, and SOD levels.

	Control	Acrylamide	P.P.	P.P. + Acrylamide
Catalase (U/mL)	3.6 ± 0.2 ^a^	1.4 ± 0.04 ^b^	3.3 ± 0.3 ^a^	3.0 ± 0.05 ^a^
SOD (U/mL)	32.7 ± 0.6 ^b^	18.3 ± 0.8 ^c^	41.6 ± 0.9 ^a^	31.2 ± 1.5 ^b^
GSH (nmol/L)	33.4 ± 0.6 ^b^	16.7 ± 1.4 ^d^	36.6 ± 2.3 ^a^	29.2 ± 1.9 ^c^
MDA (nmol/mL)	24.4 ± 1.3 ^c^	72.5 ± 1.7 ^a^	23.7 ± 2.1 ^c^	34.5 ± 1.2 ^b^

Means within the same row carrying different superscript letters a, b, c, d are significantly different at *p* < 0.05.

**Table 5:** Protective effects of pomegranate-peel extract against acrylamide-induced alterations in cytokine levels.

	Control	Acrylamide	P.P.	P.P. + Acrylamide
IL-1β (pg/mL)	92.6 ± 5 ^c^	240.4 ± 8.7 ^a^	78.2 ± 9.6 ^d^	112.8 ± 7.3 ^b^
IL-6 (pg/mL)	77.0 ± 5.7 ^d^	274.4 ± 11.2 ^a^	100.8 ± 9.6 ^c^	119.5 ± 5.7 ^b^
IL-10 (pg/mL)	118.0 ± 6.9 ^b^	77.6 ± 3.9 ^d^	147.6 ± 7.6 ^a^	112.9 ± 6.2 ^c^

Means within the same row carrying different superscript letters a, b, c, d are significantly different at *p* < 0.05.

## Data Availability

All data sets obtained and analyzed during the current study are available in the manuscript.
